# *De Novo* assembly, characterization and development of EST-SSRs from *Bletilla striata* transcriptomes profiled throughout the whole growing period

**DOI:** 10.1371/journal.pone.0205954

**Published:** 2018-10-26

**Authors:** Delin Xu, Hongbo Chen, Murat Aci, Yinchi Pan, Yanni Shangguan, Jie Ma, Lin Li, Gang Qian, Qianxing Wang

**Affiliations:** 1 Department of Medical Cell Biology, Zunyi Medical University, Zunyi, Guizhou, China; 2 Department of Soil and Crop Sciences and Institute for Plant Genomics and Biotechnology, Texas A&M University, College Station, Texas, United States of America; University of Western Sydney, AUSTRALIA

## Abstract

*Bletilla striata* is an endangered orchid that has been used for millennia as a medicinal herb, in cosmetics and as a horticultural plant. To construct the first nucleotide database for this species and to develop abundant EST-SSR markers for facilitating further studies, various tissues and organs of plants in the main developmental stages were harvested for mRNA isolation and subsequent RNA sequencing. A total of 106,054,784 clean reads were generated by using Illumina paired-end sequencing technology. The reads were assembled into 127,261 unigenes by the Trinity package; the unigenes had an average length of 612 bp and an N50 of 957 bp. Of these unigenes, 67,494 (51.86%) were annotated in a series of databases. Of these annotated unigenes, 41,818 and 24,615 were assigned to gene ontology categories and clusters of orthologous groups, respectively. Additionally, 20,764 (15.96%) unigenes were mapped onto 275 pathways using the KEGG database. In addition, 25,935 high-quality EST-SSR primer pairs were developed from the 15,433 unigenes by MISA mining. To validate the accuracy of the newly designed markers, 87 of 100 randomly selected primers were effectively amplified; 63 of those yielded PCR products of the expected size, and 25 yielded products with significant amounts of polymorphism among the 4 landraces. Furthermore, the transferability test of the 25 polymorphic markers was performed in 6 individuals of two closely related genus *Phalaenopsis* and *dendrobium*. Which results showed a total of 5 markers can successfully amplified among these populations. This research provides a comprehensive nucleotide database and lays a solid foundation for functional gene mining and genomic research in *B*. *striata*. The developed EST-SSR primers could facilitate phylogenetic studies and breeding.

## Introduction

*Bletilla striata* (Thunb.) Reichb. f. (Orchidaceae) has been widely used as a common medicinal plant and horticulture specimen in many countries for several centuries. The pseudobulbs of this traditional Chinese medicinal (TCM) herb have various medicinal properties, including hemostatic [1], anti-inflammatory, and antibacterial [2] activities, as well as the ability to reduce swelling and promote tissue regeneration [3]. In addition, previous studies also demonstrated that *B*. *striata* has anticancer and antiviral properties [4–5]. This plant not only has traditional medicinal properties but also can be used as an important ornamental or potted plant in Europe and the United States [3,6].

The increasing use of this plant has accelerated its extinction risk due to over-collection of wild populations and destruction of natural habitats. We still have very limited information about its genome. Its natural habitat is mainly in southern China, extending to the Korean peninsula and Japan. It was listed as one of the key protected medicinal plants in China more than 10 years ago (http://rep.iplant.cn/protlist?key=bletilla), even though several professional cultivation methods for *B*. *striata* had been reported for many years. However, genome studies in this plant have lagged far behind those of other orchid plants because of the lack of genomic information. The only publicly available DNA data on *B*. *striata* are 19 simple sequence repeats (SSRs) reported by Zhang (2015) [7] and Wu (2013) [8], and 205 nucleotide sequences, 141 genes and 218 proteins on the NCBI website. No transcriptome data for this important species has been reported. To facilitate functional gene discovery, genetic diversity studies, and breeding projects in *B*. *striata*, genomic or transcriptomic data is urgently needed. In recent years, advances in next-generation sequencing (NGS) and assembly algorithms have resulted in fast and deep RNA sequencing technology (RNA-Seq), which is readily available for nonmodel plants. This technology has greatly facilitated the discovery of functional genes in specific biosynthesis pathways and the development of molecular markers in large numbers in important plant species [9–13].

In this study, we sampled the pooled transcriptomes of developing seedlings, roots, leaves and pollenated capsules using Illumina paired-end sequencing technology to generate a large-scale unigene database and to develop a set of EST-SSRs. To our knowledge, this is probably the first genomic study to characterize the complete transcriptome of *B*. *striata* by analyzing a large-scale collection of transcript sequences. We expect these data to provide a valuable nucleotide database of the entire lifespan of *B*. *striata* for the purposes of functional gene mining and the identification of genetic mechanisms of secondary metabolites biosynthesis. Additionally, the newly developed EST-SSR markers may facilitate further genetic research on and breeding projects in *B*. *striata*, as well as its close species.

## Materials and methods

### Plant materials and RNA extraction

*B*. *striata* seeds were purchased from a local farmer planting medicinal herbs in Zheng’an County, Guizhou Province, China, on October 20th, 2015(28°56′N, 107°43′E). Soon after, we applied tissue culture approaches to germinate the seeds into seedlings. Four months later, we planted the seedlings in fields for growing and for the next year’s flowering. The protocorms, whole plant seedlings in tissue culture containers, whole plant seedlings transplanted to fields for two months, whole plant with seeds of seedlings transplanted annually, roots, stems, leaves and pollenated capsules were randomly collected for total RNA extraction (The pictures of different period *B*. *striata* were showed in S1 Fig). The samples’ total RNA was isolated by using TRIzol reagent (Invitrogen, Carlsbad, CA, USA) according to the manufacturer’s instructions. After digestion of residual DNA with DNase I, quality assessments for purity, integrity, and concentration were performed using 1.2% agarose gel electrophoresis and spectrophotometry (OD260/280) on a NanoDrop DU8000 instrument. Then, equal amounts of each total RNA sample were pooled to create two mixed samples for subsequent studies.

### cDNA library preparation

The RNA-Seq library was constructed using Illumina’s standard pipeline (Illumina Inc., San Diego, CA, USA). First, poly-T oligo-attached magnetic beads were used for poly-A mRNA isolation from total RNA. The mRNA was fragmented into ~200 bp length segments with a fragmentation buffer. Then, first-strand cDNA was synthesized from short fragmented mRNA by using random hexamers. AMPure XP beads were used to select for fragment size after double-stranded cDNA synthesis. Subsequently, the cDNA fragments were end-paired, A-tailed, and amplified by PCR for 15 cycles. After purification, the quality of the PCR products was determined by the Agilent 2100 Bioanalyzer, Qubit 2.0 protocol. Finally, the effective concentration (EC) of the cDNA library was precisely controlled by the Q-PCR system (EC>2 nM).

### Illumina sequencing and raw data filtering

Sequencing of the *B*. *striata* cDNA libraries was performed on the Illumina HiSeqTM 2000 platform with the PE50 approach by Suzhou GeneSci Corporation, Ltd. The resulting raw reads were automatically collected into FASTQ files (.fq). Next, the raw sequences were cleaned by removing adapter contamination sequences, ambiguous sequences containing “N” nucleotide calls in more than 10% of reads and reads in which more than 50% of bases were of low quality (Qphred≤20) to generate clean reads. All clean Illumina sequencing data have been deposited in the SRA database with the accession number SRR7058048. The assembling sequences were uploaded to TSA database with the submission number of SUB4273091.

### *De novo* assembly

The clean reads were assembled *de novo* by using the Trinity package with default parameters, which has been used successfully for many nonreference species because of its outstanding performance for data from various settings [14–16]. Furthermore, the CD-HIT package was applied with default parameters to reduce redundancy from transcripts derived from homologous genes or from different alleles of the same gene, resulting in final assembly of a full-length transcript according to the de Bruijn graph theory, which uses variable transcript splicing characteristics [17].

### Unigene annotation and coding sequence (CDS) prediction

In this study, to functionally annotate the assembled unigenes, NCBI BLAST 2.2.28+ was employed for annotation using public databases, including the NCBI nonredundant protein sequences (NR), NCBI nucleotide sequences (NT), Kyoto Encyclopedia of Genes and Genomes (KEGG), Swiss-Prot protein, and Protein family (Pfam) databases, each with an E-value = 1e-5, and the euKaryotic Ortholog Groups (KOG) database with an E-value = 1e-3. In addition, Blast2GO was applied to the unigene sequences for Gene Ontology (GO) annotation using the NR and Pfam databases. Subsequently, to predict coding sequences, we first compared all unigenes against the Nr and Swiss-Prot protein databases using BLAST with an E-value = 1e-5. Then, the open reading frame (ORF) information from the BLAST hit unigenes was fetched directly. The nonhit unigenes and failed predictions (but successfully matched unigenes) were predicted by ESTScan (version 3.0.3) software.

### EST-SSR mining and primer design

MISA (MIcroSAtellite Identification Tool) and Primer 3.0 were employed for mining potential EST-SSR loci and for designing primers, respectively. The repeat number thresholds for EST-SSR marker selection were 10 for mono-nucleotide repeats, 6 for di-nucleotide, and 5 repeat units for tri-, tetra-, penta-, and hexanucleotides. The maximum interruption between two markers in a compound microsatellite was 100 bp. The EST-SSR primers were designed using the following standards: (i) annealing temperature (*T*_m_) from 57°C to 63°C, (ii) primer size from 18 bp to 27 bp, with an optimal size of 20 bp, (iii) *T*_m_ max difference between forward and reverse primer less than 2°C, (iv) predicted product size range 100–300 bp, (v) three primer pairs per marker loci, and with other parameters set to default values.

### Amplification and validation of developed EST-SSR markers

A total of 100 EST-SSR primer pairs were randomly selected for synthesis and for further validation using 4 different landraces in *B*. *striata* (S1 Table). Genomic DNA was extracted from tender leaves by the CTAB method. Then, DNA quality and quantity were determined by 0.8% agarose gel and NanoDrop DU8000 spectrophotometry. The DNA concentration was adjusted to 20 ng/μl for subsequent PCR amplification of SSR markers.

To test the amplification efficiency and polymorphism of the selected markers, PCR was performed in a total volume of 10 μl, containing 5 μl 2×Master MIX (TSINGKE Biological Technology Development Co., Ltd., Chengdu, China), 1 μl DNA template, 0.5 μl of each primer (100 μM), and 3 μl ddH_2_O. PCR amplification was accomplished using the following conditions: predenaturation at 94°C for 3 min; followed by 35 cycles of denaturation at 94°C for 30 sec, annealing at 58–62°C (depending on the primer) for 30 sec, and extension at 72°C for 30 sec; a final 7 min extension at 72°C; and then a hold at 16°C. The obtained PCR products were separated on a 6% PAGE gel at 120 V for 3 h and then visualized by AgNO_3_ solution. The DNA ladder, B500331 Marker B (Sangon Biological Technology Development Co., Ltd, Shanghai, China), was used as a size standard for the bands. Further, to evaluate the transferability of 25 polymorphic markers, six individuals from two related orchids *Phalaenopsis* and *dendrobium* (3 individuals for each population, S2 Table) were amplified by the same PCR protocol.

## Results

### Sequencing and assembly

**Fig 1:**
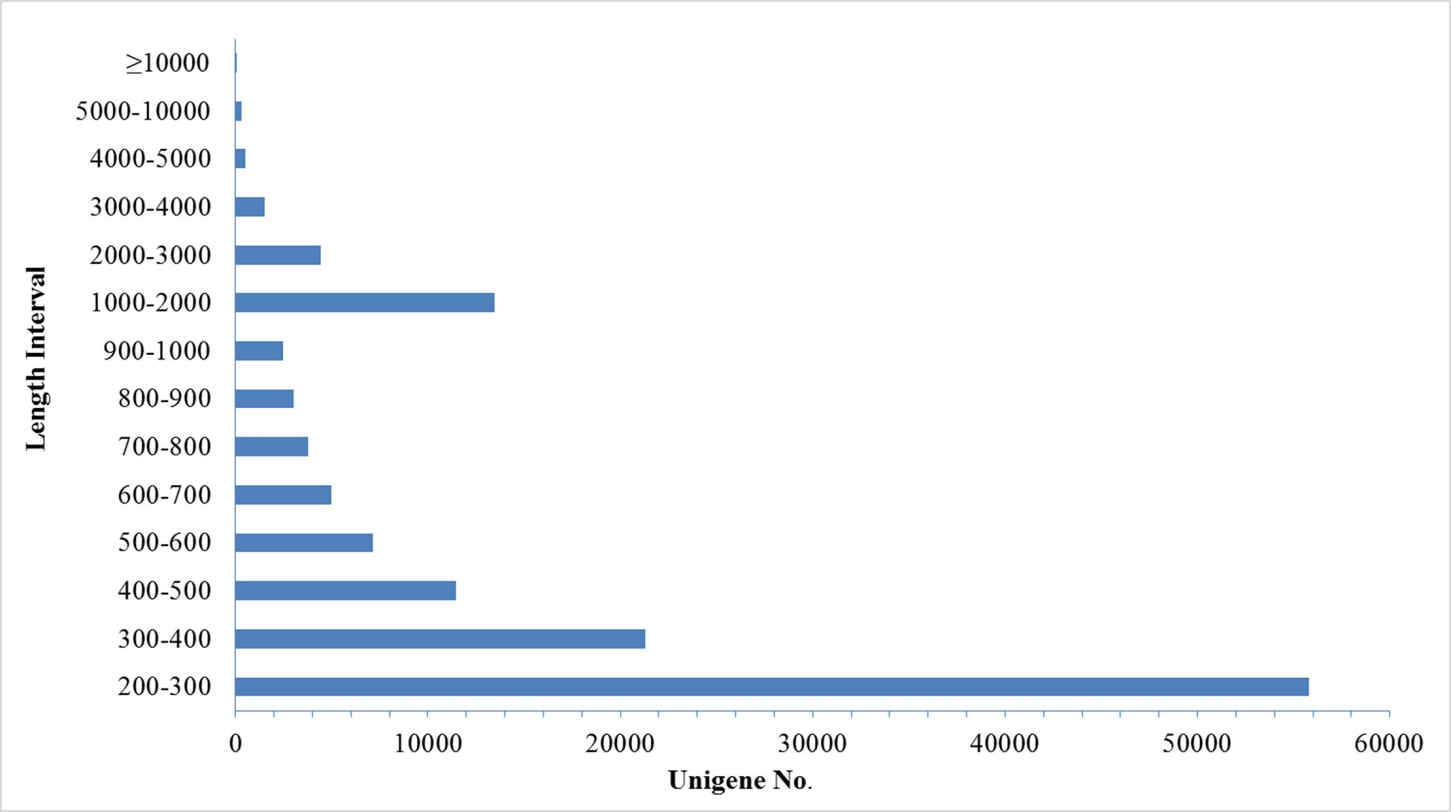
Length distribution of assembled unigenes. The x-axis indicates the number of unigenes, and the y-axis indicates the length range.

We used normalized RNA pools, from developing roots, stems, leaves and pollenated capsules of protocorms, cultured seedlings, newly planted field seedlings, and transplanted adult plants for sequencing to enhance the likelihood of harvesting expressed genes and to obtain global developmental transcriptomes for the whole life of the plant. By merging sequencing results of the two duplicates, a total of 109,714,290 paired-end raw data reads were generated from the Illumina HiSeq Sequencing 2000 platform, with a GC content of 47.87%. The sequence quality assessment showed that Q20 and Q30 were 95.69% and 91.49%, respectively. After filtering, 106,054,784 clean reads were generated in total (Table 1). Further, 127,261 overlapping unigenes were identified by the Trinity software package. The length statistics and feature analysis of these unigenes showed a minimum, maximum and mean length of 201 bp, 21,950 bp, and 612 bp, respectively, an N50 of 957 bp, and an N90 of 249 bp (Table 1). Additionally, of these unigenes, 88,532 (68.03%) were 200–500 bp, 21,395 (16.44%) were 501–1000 bp, 13,459 (10.34%) were 1001–2000 bp, and only 6751 unigenes (5.19%) were more than 2000 bp in length. Obviously, there was a trend of a reduced number of unigenes with an increase in the assembly length (Fig 1).

**Table 1:** Summary of the analysis of the de novo assembly and EST-SSR identification.

Category	Items	Description
Raw reads	Total raw reads	109,714,290
Clean reads	Total clean reads	106,054,784
	Total clean nucleotides (nt)	13.26 G
	Q20 (%)	95.69%
	Q30 (%)	91.49%
	GC content (%)	47.87%
Unigenes	Total sequences	127,261
	Total sequence bases (Kb)	79,698
	Largest (bp)	21,950
	Smallest (bp)	201
	Average (bp)	612
	N50 (bp)	957
	N90 (bp)	249
	Annotated	67,494
EST-SSR	Total number of tested sequences	127,261
	Total size of tested sequences (Kb)	79,698
	Total number of identified SSRs	18,335
	Number of SSR-containing sequences	15,433
	Number of sequences containing more than one SSR	2,427
	Number of SSRs present in compound formation	778

### Gene functional annotation and metabolic pathway analysis

To functionally annotate the assembled unigenes, all unigene sequences were blasted against the seven databases (see Materials and methods), resulting in the successful annotation of 49,355 (37.93%), 34,751 (26.70%), 20,764 (15.96%), 40,109 (30.82%), 40,920 (31.44%), and 24,615 (18.91%) unigenes, respectively. As a result, a total of 67,494 (51.86%) unigenes were annotated in at least one of the seven databases mentioned above (S3 Table).

**Fig 2:**
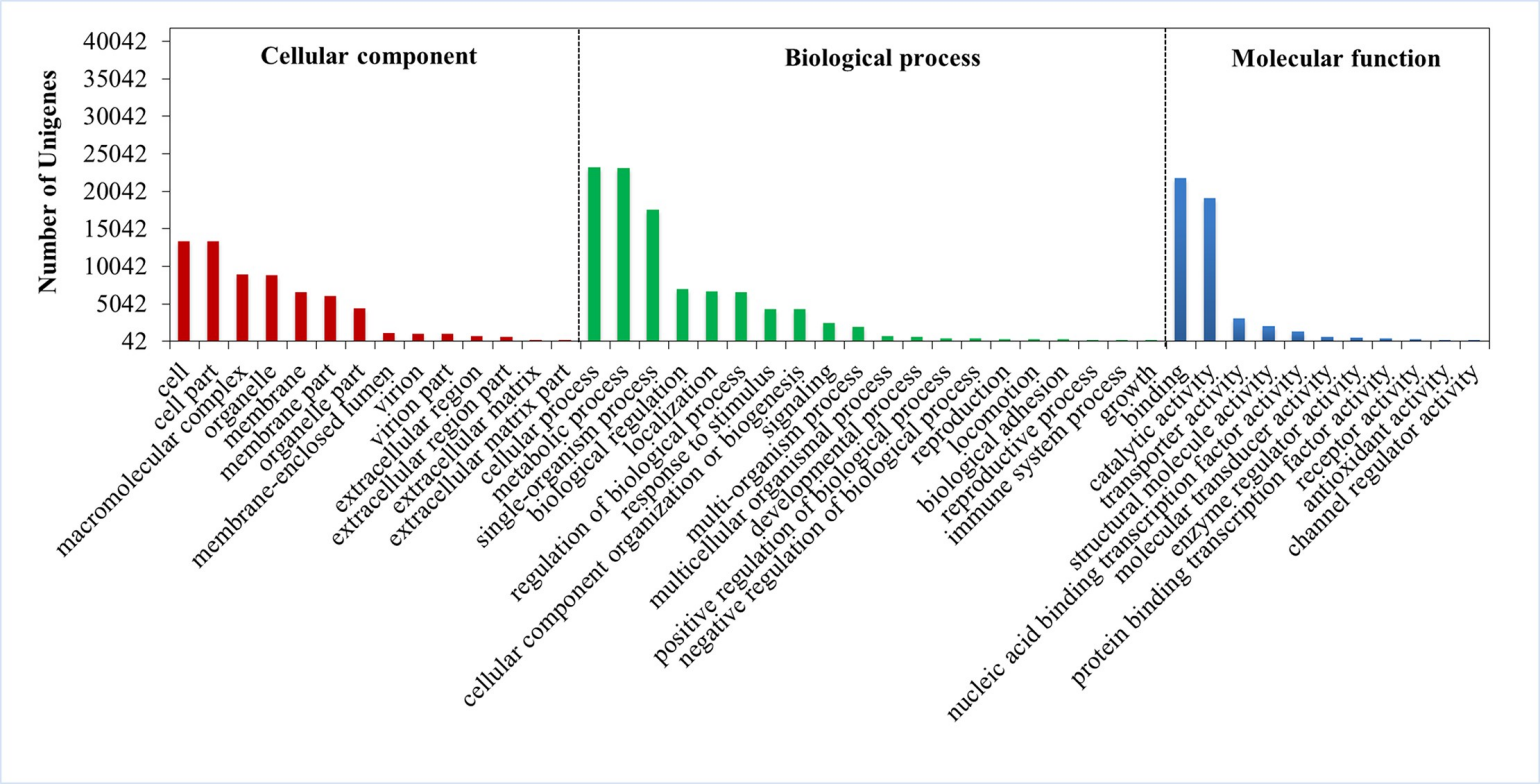
GO classification of unigenes in the categories of Biological Process, Cellular Component, and Molecular Function. The left *y*-axis shows the number of unigenes annotated in each subcategory. The abscissa axis represents the subterms of Biological Process, Cellular Component, and Molecular Function, three different GO categories.

GO functional annotation was obtained by the Blast2GO Program, which, when combined with InterProScan, an internationally standardized gene function classification system with dynamically updating vocabulary, can exactly define the characteristics of genes and their products [18–19]. The results showed that a total of 41,818 (32.13%) unigenes fell into three main ontologies, including Biological Process, Cellular Component, and Molecular Function (Fig 2). With respect to the Biological Process category, cellular process (total hit 23,257 unigenes, 55.61%), metabolic process (23,124 unigenes, 55.30%), and single-organism process (17,570 unigenes, 42.02%) were the three main subcategories. In the Cellular Component category, cell (13,310 unigenes, 31.83%) and cell part (13,302 unigenes, 31.81%) were the dominant terms, and in the Molecular Function category, binding (21,760 unigenes, 52.04%) and catalytic activity (19,082 unigenes, 45.63%) were the most enriched subgroup. However, the GO functional annotation results covered only a fraction of the assembled unigenes annotated in GO categories. This may be due mainly to the enormous absence of genetic information and the lack of gene resources.

**Fig 3:**
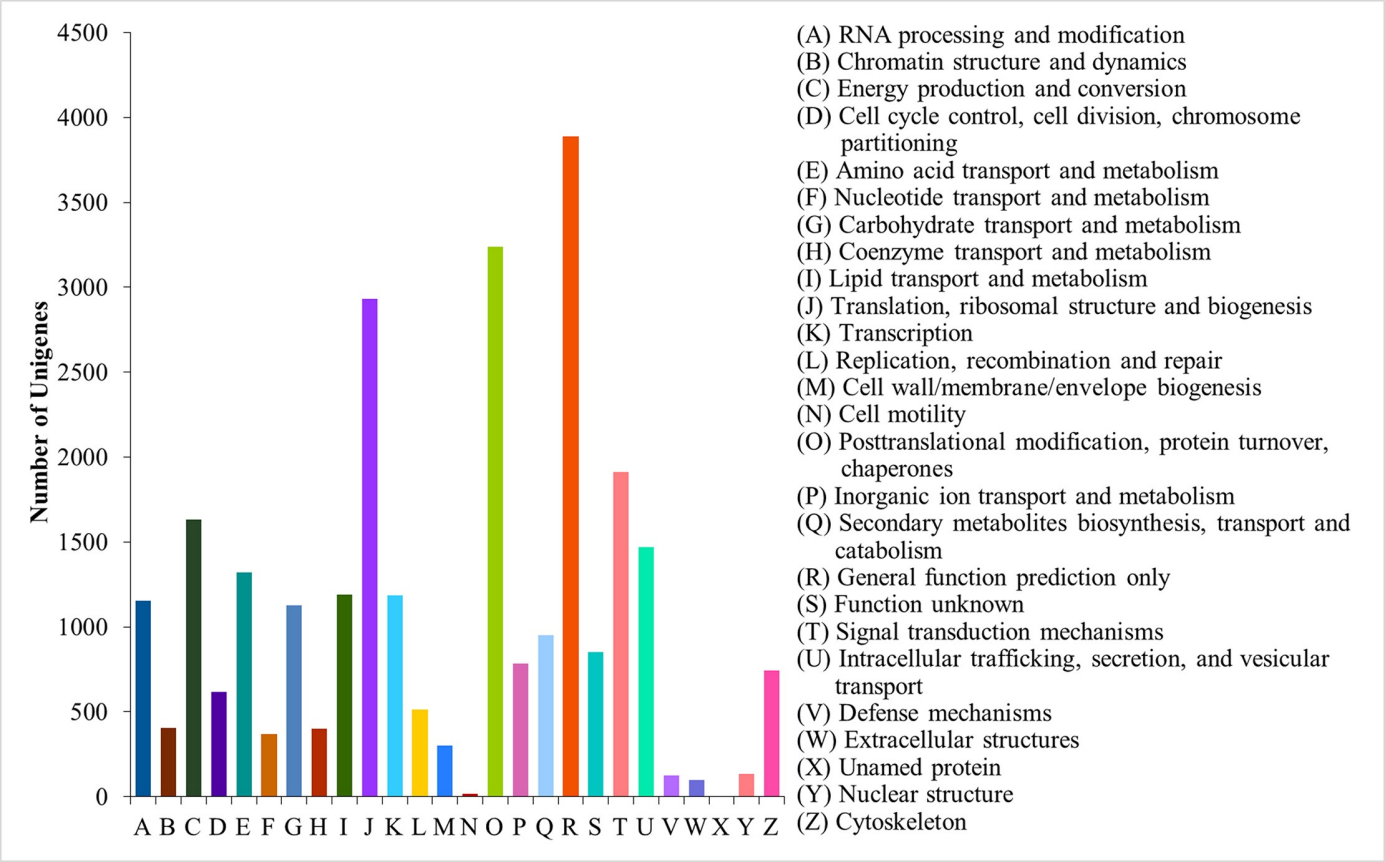
KOG function classification of assembled unigenes. The *x*-axis represents the 26 KOG groups. The *y*-axis shows the number of unigenes in each KOG functional category.

KOG annotation was completed by running BLAST (*E*-value = 1e-3) against the KOG databases, resulting in 24,615 (18.91%) unigenes being categorized into 26 groups (Fig 3). Analysis of the KOG annotation results showed that the R (3,889 unigenes, 15.80%), O (3,240 unigenes, 13.16%), and J (2,931 unigenes, 11.91%) categories were the three largest primary groups, representing “General function prediction only”, “Posttranslational modification, protein turnover, chaperones”, and “Translation, ribosomal structure and biogenesis”, respectively.

Further, to predict metabolic pathways in the current transcriptome, all unigenes were blasted against the KEGG database with a threshold *E*-value of 1e-5. In total, 20,764 (15.96%) unigenes were classified into 275 different pathways (S4 Table). The results showed that the three top categories were Translation (2,831 unigenes, 13.63%), Carbohydrate metabolism (2,250 unigenes, 10.84%) and Signal transduction (2,097 unigenes, 10.10%), representing genes involved in genetic information processing, metabolism and environmental information processing, respectively. Moreover, KEGG pathways annotation also indicated that many unigenes were involved in important pathways such as Plant-pathogen interaction (299 unigenes), Plant hormone signal transduction (246 unigenes), Circadian rhythm–Plant (66 unigenes), RNA transport (544 unigenes), RNA polymerase (111 unigenes), DNA replication (106 unigenes), Cell cycle pathway (278 unigenes), etc. In addition, a total of 233, 39, 102, 63 and 29 genes were related to Phenylpropanoid biosynthesis (ko00940), Flavonoid biosynthesis (ko00941), Terpenoid biosynthesis (ko00900), Ubiquinone and Other terpenoid-quinone biosynthesis (ko00130), and Diterpenoid biosynthesis (ko00904), respectively. Based on current knowledge, these important metabolic pathways could lead to pathways for the synthesis of antioxidant micromolecules in *B*. *striata*, which possess considerable bioactivity as antioxidants ^[^^17^^]^. Many unigenes were found to be in the Cell cycle (ko04110), Plant hormone signal transduction (ko04075), DNA replication (ko03030), MAPK signaling pathway (ko04010) and Purine metabolite (ko00230) categories. These pathways are very significant not only in plant growth and development but also in closely related synthetic processes for primary and secondary products.

### Coding sequence (CDS) prediction

To predict the coding sequences, we first blasted all unigenes against the NR and Swiss-Prot protein databases and then applied ESTScan to predict the ORF of unigenes successfully matched by BLAST. As a result, a total of 51,214 matched coding sequences were successfully translated into protein sequences (sequence direction is 5’→3’), and another 58,515 coding sequences were predicted by ESTScan 3.0.3. Further, the length distribution analysis showed that as the length of the CDS increased, the number of sequences was reduced; the CDS lengths ranged from 27 to 15,381 bp, with an average length of 200–300 bp (14,009 unigenes, 27.35%). The largest number of CDSs from ESTScan 3.0.3 was distributed in the 100–300 bp range (34,859 genes, 59.57%), and the lengths ranged from 51–13,263 bp (S1 Fig). The maximum length of an encoded protein sequence was 5,127 bp (c70543-g1).

### Microsatellite locus analysis and SSR marker development

Additionally, 18,335 SSRs were identified in 15,433 unigenes, not including compound SSRs. Based on the analysis, mono- repeats were the largest category and accounted for 49.78% (9,128 SSRs) of the unigenes, followed by di- (4,884 SSRs, 26.64%), and tri- (4,166 SSRs, 22.72%) repeats. In addition, the quantitative statistics for mono-, di-, tri- and tetra- repeats showed that the most abundant motifs were A/T (8,996, 98.55%), AG/CT (3,286, 67.28%), AAG/CTT (1,020, 24.48%), and AAAT/ATTT (30, 25.86%), respectively (Table 2). Furthermore, a total of 25,935 EST-SSR markers were successfully designed for 8,645 SSR loci by online Primer 3.0 (S5 Table).

**Table 2:** Characterization of SSR analysis in assembled unigenes.

Repeat type	SSR number	Proportion (%)	Distribution density (1/kb)	Number of motif	Main repeat motif
Mono-	9,128	49.78	1/8.87 kb	2	A/T (98.55%)
Di-	4,884	26.64	1/16.57 kb	4	AG/CT (67.28%)
Tri-	4,166	22.72	1/19.42 kb	10	AAG/CTT (24.48%)
Tetra-	116	0.63	1/697.62 kb	28	AAAT/ATTT (25.86%)
Penta-	19	0.10	1/4259.13 kb	15	AAGAG/CTCTT (15.78%)
Hexa-	22	0.12	1/3678.34 kb	21	-
Total	18,335	100	1/4.41 kb	78	-

### Validation of designed EST-SSR markers

To evaluate the amplification efficiency and polymorphism of designed markers, we randomly selected 100 EST-SSR markers (S6 Table) and amplified them in 4 different landraces in *B*. *striata*. The PCR results showed that a total of 87 primer pairs were successfully amplified; of those, 63 yielded PCR products of the expected size and 25 primer pairs showed obvious polymorphism in this study (S2 Fig, S7 Table). Cross-species amplification results showed that 2 markers (ZYBS-1, ZYBS-52) were consistently amplified in both *Phalaenopsis* and *dendrobium*, 1 marked as ZYBS-14 only has bands in *Phalaenopsis*, and 2 loci (ZYBS-18, ZYBS-60) were amplified only in *dendrobium* (S4 Fig).

## Discussion

### Characterization of *B*. *striata* Transcriptome

In the present study, a total of 106,054,784 clean reads were successfully generated from 109,714,290 raw reads by Illumina RNA sequencing technology for *B*. *striata*, with 95.69% Q20 and 91.49% Q30 quality levels. Further, 127,261 unigenes were assembled with an N50 of 957 bp and a mean length of 612 bp. Global developmental transcriptome data for *B*. *striata* has been generated by this study, which will facilitate further understanding and genomic research for this important medicinal plant, especially for functional gene mining and the illustration of pathways for chemicals synthesis, as well as the development of molecular markers and functional genomics. These data may also serve as a reference for other closely related species, such as *Phalaenopsis* and *dendrobium* et al.

An abundance of assembled *B*. *striata* unigenes were successfully annotated in the seven listed public databases (S2 Table). However, the annotation ratio in each database indicated that only a small portion of the assembled genome was annotated, possibly because of the limited annotation resources for the *Bletilla* genus and its related species in current databases. Therefore, the unigenes that could not be annotated can still provide valuable information for further genetic study of *B*. *striata* and closely related species.

As far as we known, *B*. *striata* mainly has the phytochemical of polysaccharide, phenols, bibenzyls, phenanthrenes, triterpenoids and its saponins, steroids and its saponins, and which has been proven to related to a series of medicinal properties. For depth understanding between these components and its possible reflect genes, A total of 41,818 (32.13%) and 20764 (15.96%) unigenes were assigned to GO and KEGG categories respectively. In addition, we found GO:0030246, GO:0033036, GO: 0097367, GO:1901476, GO:0005975 and GO:1901135, GO:0008643, GO:1901264 may be related to the polysaccharide biosynthesis process of *B*. *striata*. And moreover, from KEGG analyses, a total of 2,250 unigenes (10.84%) were related to the Carbohydrate metabolism. But for the best of our knowledge, the exact relationship between these genes and its medical value is still unknown. Therefore, these results not only provide a foundation for gene function research of *B*. *striata* but also indicated that the direction for further studies of this important medicinal plant should focus on some essential primary and secondary metabolites and the medicinal functions of this TCM herb.

### Distribution and Validation of EST-SSRs

As is well known, EST-SSR molecular markers are very significant to the study of population structure, genetic diversity and genetic linkage mapping [20]. In this study, 18,335 potential SSRs were identified from 15,433 unigenes, not including compound SSRs. The EST-SSR frequency in our study (14.09%) is in the high end of the range found for other angiosperms (1.5–19.25%) [21–24] and has a high distribution density of 4.41 kb per SSR loci. In *B*. *striata*, the dinucleotide repeats (4,884 SSRs, 26.64%) were the most abundant type (except for the mono-), followed by tri- (4,166 SSRs, 22.72%).

In addition, the AG/CT (3,286, 67.28%) motif was the most abundant of the dinucleotide repeats, as it is in *N*. *sericea* [25]. This may be because AG/CT are represented in CUC and UCU, which translate into the Ala and Leu amino acids, respectively, which are the most abundant amino acids in proteins [26–27]. Of the tri-nucleotides, AAG/CTT (1,020, 24.48%) was the most common motif, followed by AGG/CCT (653, 15.67%), CCG/CGG (607, 14.57%), and AAC/GTT (478, 11.47%). By contrast, ACT/AGT (29, 0.70%) was the rarest motif. Interestingly, previous studies on some other monocots, such as *Hordeum*, *Sorghum* and corn [28–30], showed that the CCG/CGG motif was the predominant trinucleotide repeat, but this repeat is very rare in some dicots [31–32]. These differences among *B*. *striata* and other monocots and dicots might be due mainly to differences in the GC content of the transcriptomes [25].

Furthermore, 25,935 EST-SSR markers were successfully designed for 8,645 EST-SSR loci in the *B*. *striata* transcriptome (S5 Table). Based on the validation of 100 randomly selected EST-SSR primer pairs, the amplification rate of our designed EST-SSR markers was 87%, of which 63 yielded PCR products of the expected size and 25 showed obvious polymorphism (S3 Fig). Relatively speaking, the ratios of amplification and generation of polymorphic EST-SSR markers were high compared to those found in the development of EST-SSR markers in other species [22]. The transferability test showed only 3 and 4 primer pairs can successfully amplified in *Phalaenopsis* and *dendrobium*, which has been demonstrated that although across-species amplification is an inefficiency but a time-effective and cost-saving way for molecular markers development [33–35]. Further research on a larger population of *B*. *striata* will be needed to confirm the information from these amplified EST-SSR markers.

## Conclusions

To the best of our knowledge, this research is the first to study the global transcriptome of all developmental stages of *B*. *striata*. The *de novo* assembled unigenes and related group results in this study provided a key genomic resource for further research into functional genomics and proteomics in this species. The EST-SSR markers developed will serve as a valuable resource for gene function characterization, comparative genomics, and molecular-assisted selection (MAS) for this important plant, as well as other closely related species.

## Supporting information

S1 Fig Pictures of sequencing materials in 4 stages. A-D represented to the life period of protocorms, whole plant seedlings in tissue culture containers, whole plant seedlings transplanted to fields for two months, whole plant with seeds of seedlings transplanted annually, respectively.
(TIF)

S2 Fig Length distribution of Coding Sequence (CDS) predication by blast in NR and Swiss-prot protein databases (A), or by EST-scan3.0.2 version (B).
(TIF)

S3 Fig PAGE electrophoresis results of selected 100 EST-SSR markers. (DOCX)

S4 Fig Cross-amplification results of 25 polymorphic markers in six individuals represented two related species Phalaenopsis and Dendrobium. (DOC)

S1 Table Landraces information in validation approach of random selected EST-SSR markers. (DOCX)

S2 Table Cultivars information in cross-species validation approach of 25 polymorphic EST-SSR markers. (DOCX)

S3 Table Summary of annotation results in different databases of all Unigenes. (DOCX)

S4 Table Characteristics of KEGG pathways for assembled *B*. *striata* unigenes. (XLSX)

S5 Table Basic information of all successfully designed EST-SSR primer pairs in *B*. *striata*. (XLSX)

S6 Table Basic information of random selected 100 Primer pairs. (XLSX)

S7 Table Basic information of 25 polymormic primer pairs. (XLSX)
