# Supplementary figures and images for: De Novo assembly, characterization and development of EST-SSRs from Bletilla striata transcriptomes profiled throughout the whole growing period
Source: PLoS One. 2018 Oct 26;13(10):e0205954. doi: 10.1371/journal.pone.0205954 (PMC6203367; doi:10.1371/journal.pone.0205954)

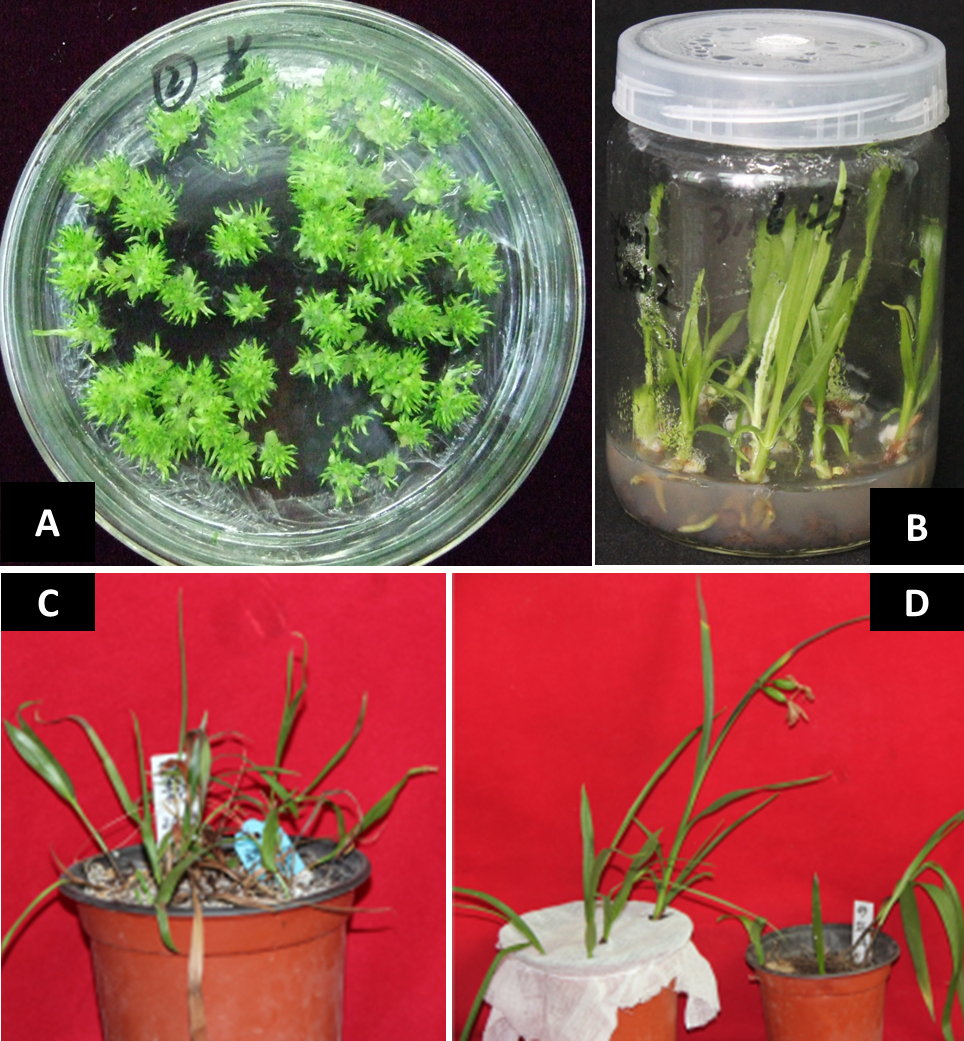

Supplement: S1 Fig — A-D represented to the life period of protocorms, whole plant seedlings in tissue culture containers, whole plant seedlings transplanted to fields for two months, whole plant with seeds of seedlings transplanted annually, respectively. (TIF) [file pone.0205954.s001.tif]

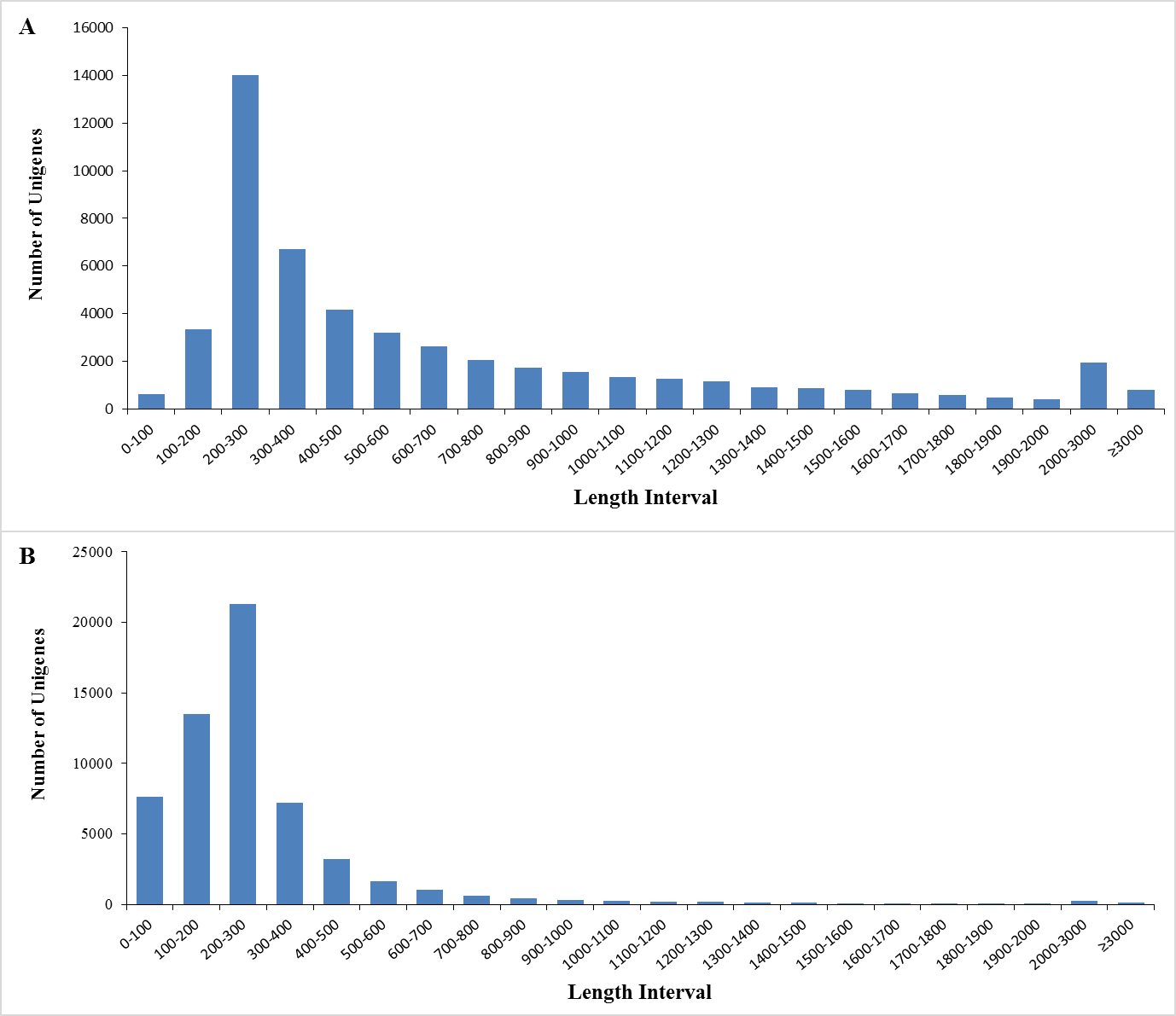

Supplement: S2 Fig — Length distribution of Coding Sequence (CDS) predication by blast in NR and Swiss-prot protein databases (A), or by EST-scan3.0.2 version (B). (TIF) [file pone.0205954.s002.tif]
