# Supplementary material for: De Novo assembly, characterization and development of EST-SSRs from Bletilla striata transcriptomes profiled throughout the whole growing period
Source: PLoS One. 2018 Oct 26;13(10):e0205954. doi: 10.1371/journal.pone.0205954 (PMC6203367; doi:10.1371/journal.pone.0205954)
Supplement: S3 Fig — (DOCX) [file pone.0205954.s003.docx]

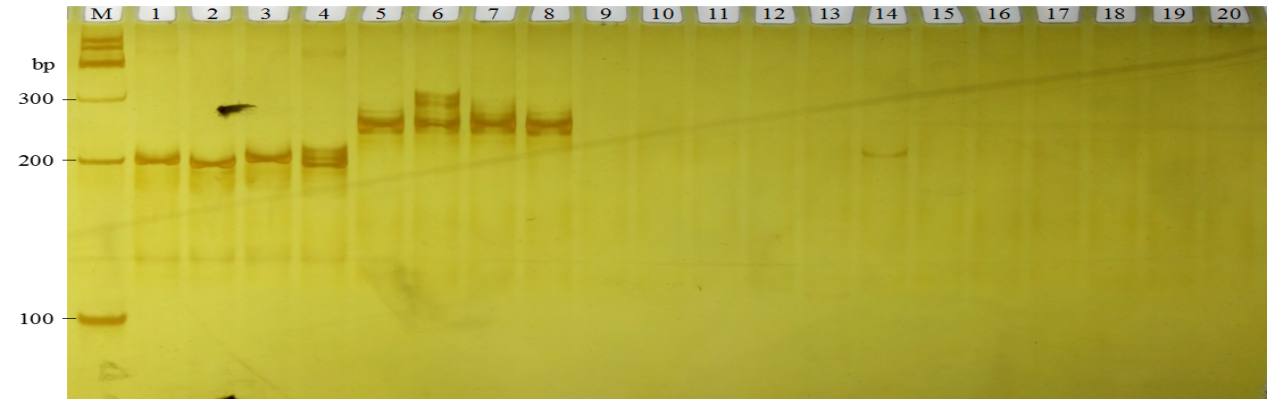


**A** 1-4: ZYBS-1; 5-8: ZYBS-2; 9-12: ZYBS-3; 13-16: ZYBS-4; 17-20: ZYBS-5


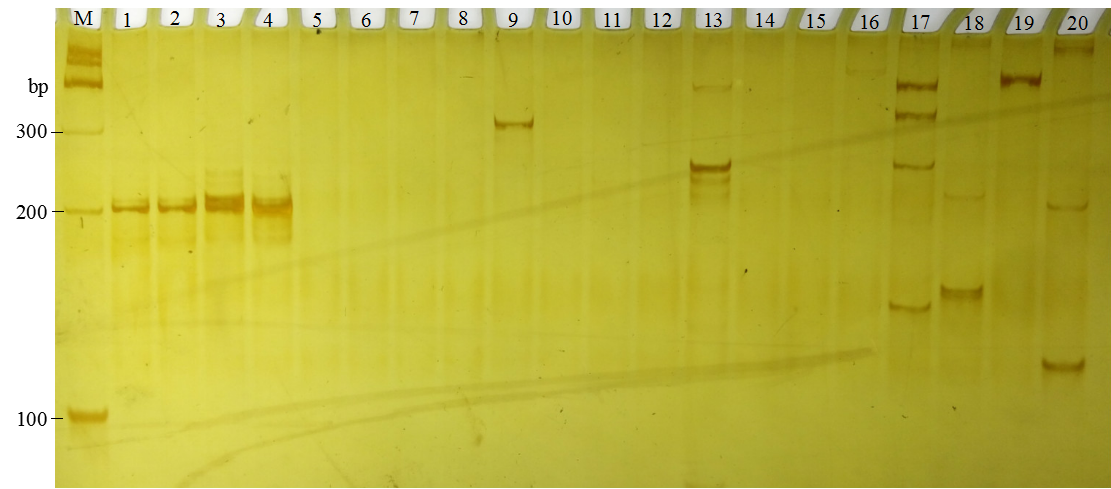


**B** 1-4: ZYBS-6; 5-8: ZYBS-7; 9-12: ZYBS-8; 13-16: ZYBS-9; 17-20: ZYBS-10


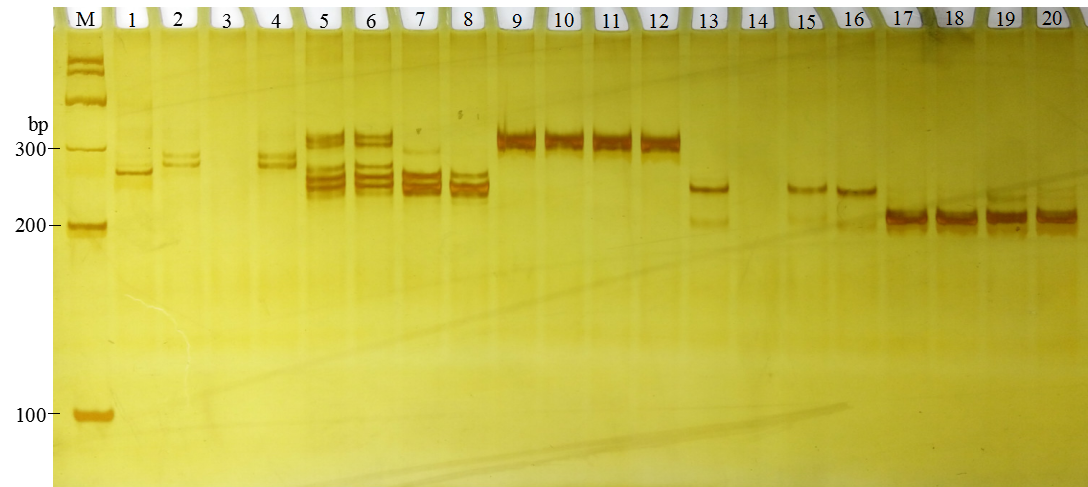


**C** 1-4: ZYBS-11; 5-8: ZYBS-12; 9-12: ZYBS-13; 13-16: ZYBS-14; 17-20: ZYBS-15


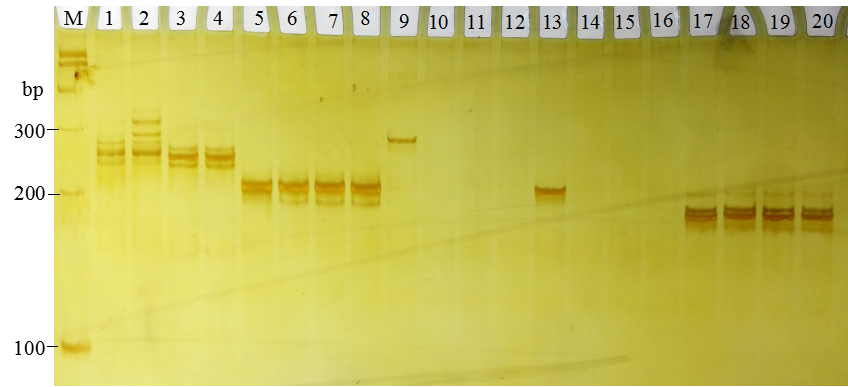
**D** 1-4: ZYBS-16; 5-8: ZYBS-17; 9-12: ZYBS-18; 13-16: ZYBS-19; 17-20: ZYBS-20
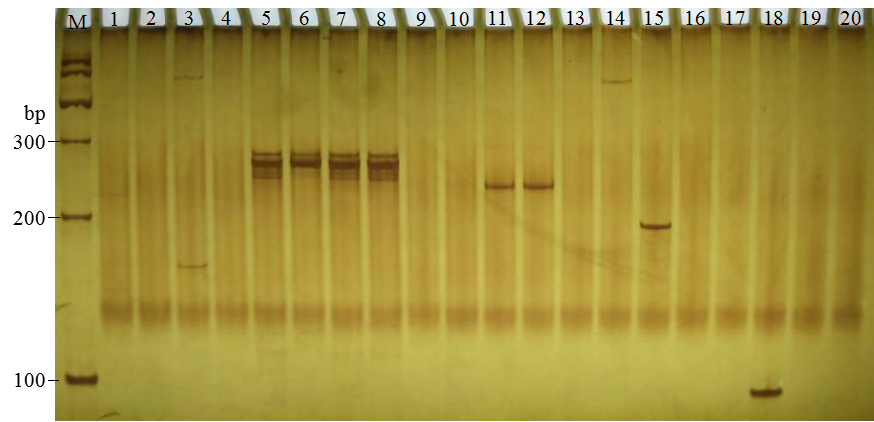


**E** 1-4: ZYBS-21; 5-8: ZYBS-22; 9-12: ZYBS-23; 13-16: ZYBS-24; 17-20: ZYBS-25


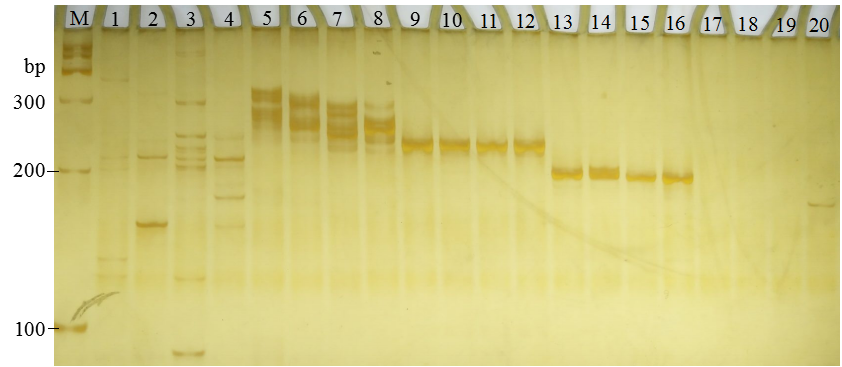


**F** 1-4: ZYBS-26; 5-8: ZYBS-27; 9-12: ZYBS-28; 13-16: ZYBS-29; 17-20: ZYBS-30


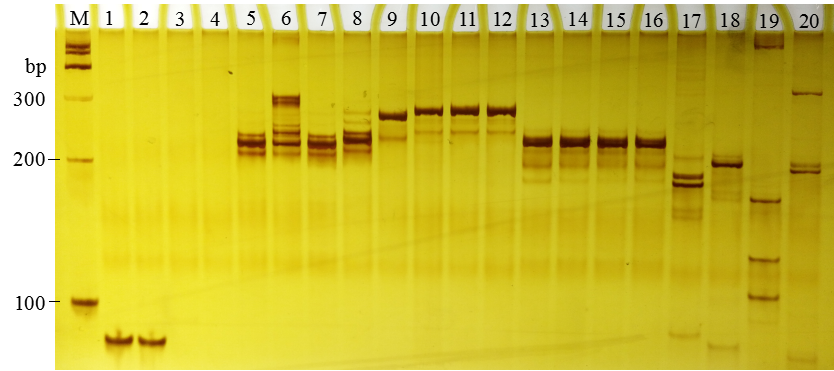
**G** 1-4: ZYBS-31; 5-8: ZYBS-32; 9-12: ZYBS-33; 13-16: ZYBS-34; 17-20: ZYBS-35
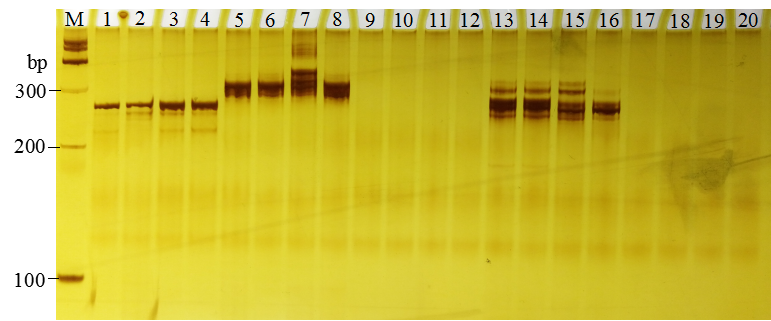


**H** 1-4: ZYBS-36; 5-8: ZYBS-37; 9-12: ZYBS-38; 13-16: ZYBS-39; 17-20: ZYBS-40
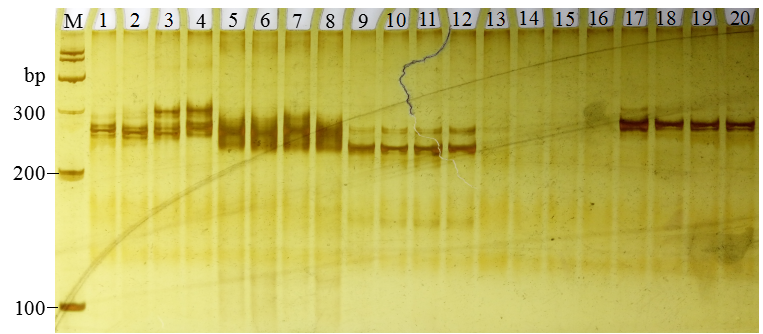


**I** 1-4: ZYBS-41; 5-8: ZYBS-42; 9-12: ZYBS-43; 13-16: ZYBS-44; 17-20: ZYBS-45
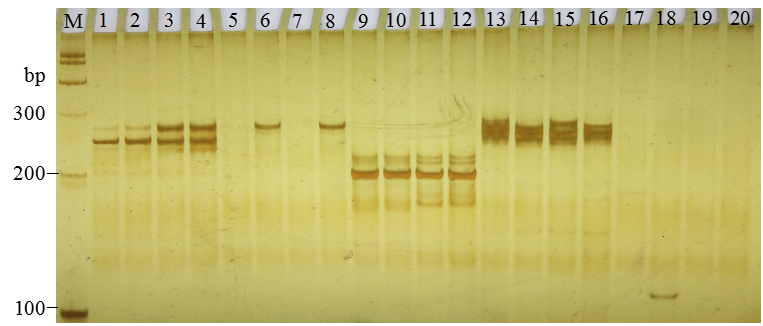


**J** 1-4: ZYBS-46; 5-8: ZYBS-47; 9-12: ZYBS-48; 13-16: ZYBS-49; 17-20: ZYBS-50
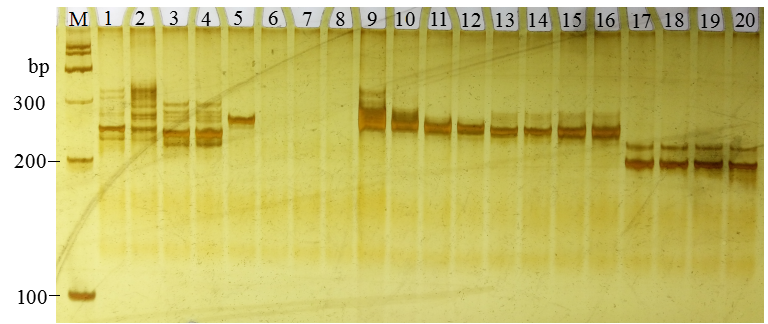


**K** 1-4: ZYBS-51; 5-8: ZYBS-52; 9-12: ZYBS-53; 13-16: ZYBS-54; 17-20: ZYBS-55


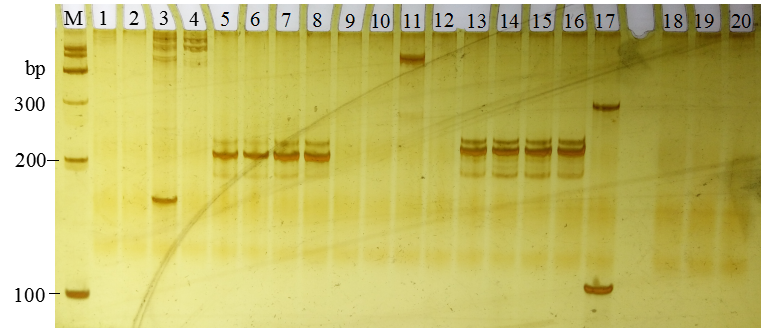


**L** 1-4: ZYBS-56; 5-8: ZYBS-57; 9-12: ZYBS-58; 13-16: ZYBS-59; 17-20: ZYBS-60
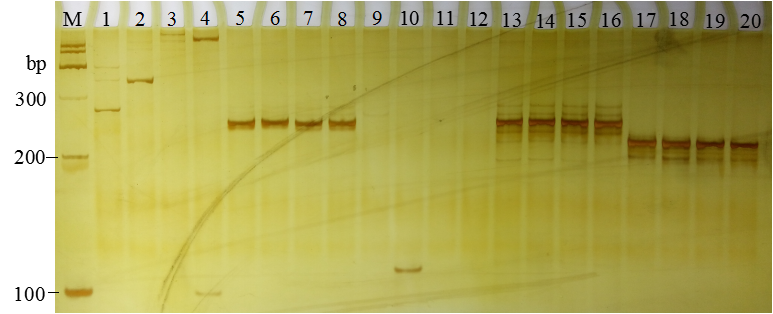


**M** 1-4:ZYBS-61; 5-8:ZYBS-62; 9-12:ZYBS-63; 13-16:ZYBS-64; 17-20:ZYBS-65
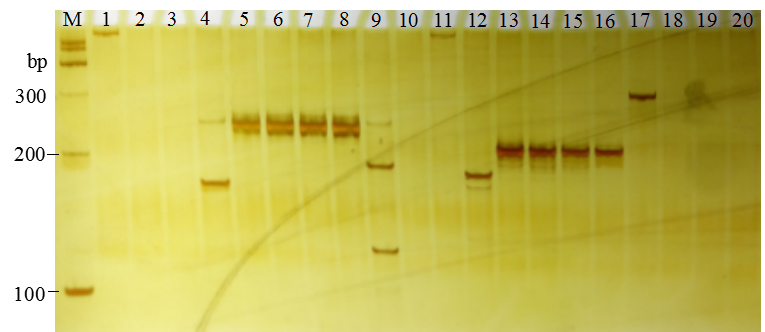
 **N** 1-4:ZYBS-66; 5-8:ZYBS-67; 9-12:ZYBS-68; 13-16:ZYBS-69; 17-20:ZYBS-70


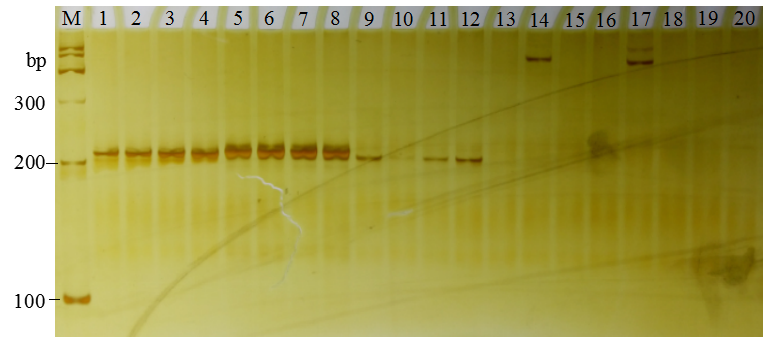


**O** 1-4: ZYBS-71; 5-8: ZYBS-72; 9-12: ZYBS-73; 13-16: ZYBS-74; 17-20: ZYBS-75
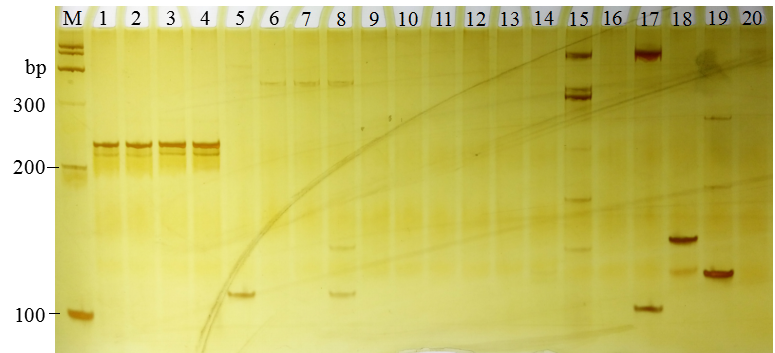


**P** 1-4: ZYBS-76; 5-8: ZYBS-77; 9-12: ZYBS-78; 13-16: ZYBS-79; 17-20: ZYBS-80
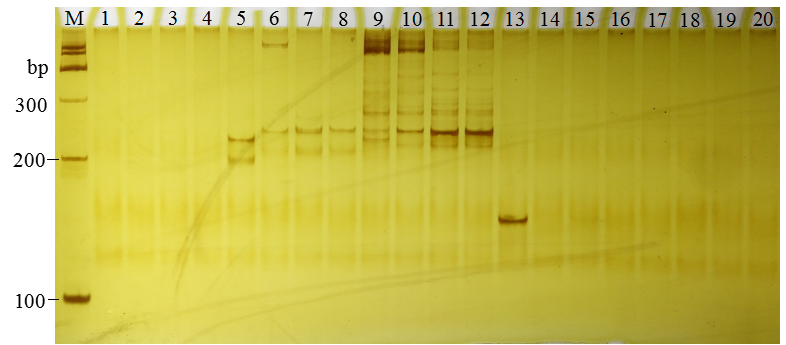


**Q** 1-4: ZYBS-81; 5-8: ZYBS-82; 9-12: ZYBS-83; 13-16: ZYBS-84; 17-20: ZYBS-85
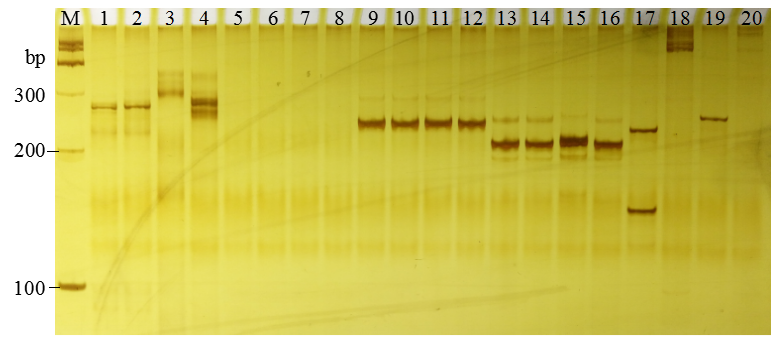


**R** 1-4: ZYBS-86; 5-8: ZYBS-87;9-12: ZYBS-88;13-16: ZYBS-89;17-20: ZYBS-90
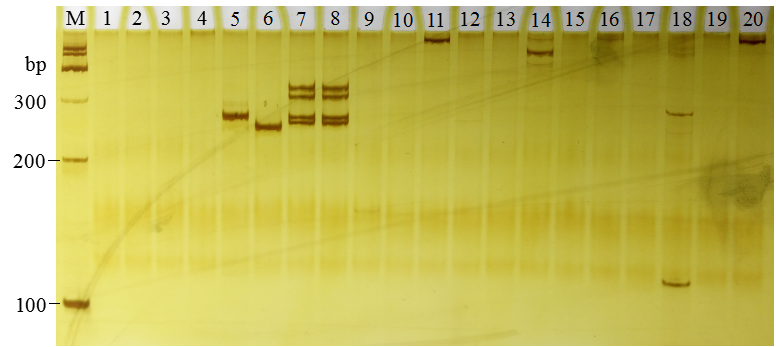


**S** 1-4: ZYBS-91; 5-8: ZYBS-92; 9-12: ZYBS-93; 13-16: ZYBS-94; 17-20: ZYBS-95
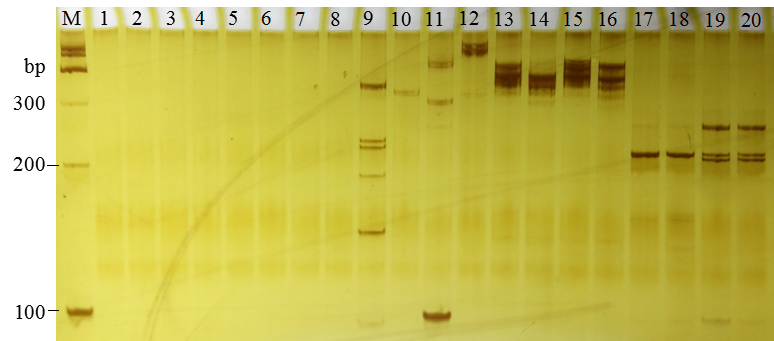


**T**1-4: ZYBS-96; 5-8: ZYBS-97; 9-12: ZYBS-98; 13-16: ZYBS-99; 17-20: ZYBS-100

S3 Figure. PAGE electrophoresis results of selected 100 EST-SSR markers. For each picture, from 1^st^ to 4^th^, 5^th^ to 8^th^, 9^th^ to 12^th^, 9^th^ to 12^th^, 13^th^ to 16^th^, 17^th^ to 20^th^ lanes, the template genotypes corresponding to the landraces of HH, BH, ZS and LS, respectively.
