# Supplementary material for: De Novo assembly, characterization and development of EST-SSRs from Bletilla striata transcriptomes profiled throughout the whole growing period
Source: PLoS One. 2018 Oct 26;13(10):e0205954. doi: 10.1371/journal.pone.0205954 (PMC6203367; doi:10.1371/journal.pone.0205954)
Supplement: S4 Fig — (DOC) [file pone.0205954.s004.doc]

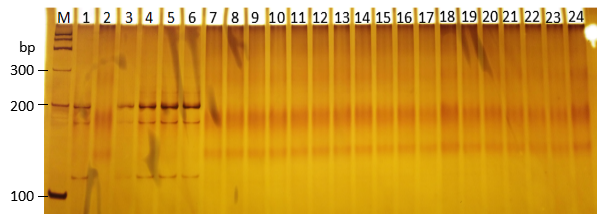


**A**  1-6: ZYBS-1; 7-12: ZYBS-9; 13-18: ZYBS-11; 19-24: ZYBS-12

**
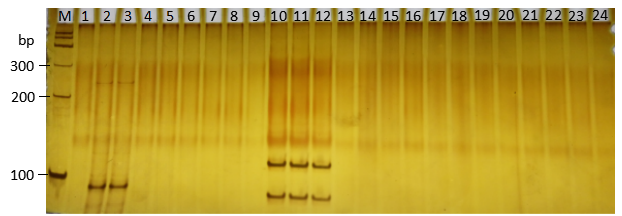
**

**B** 1-6: ZYBS-14; 7-12: ZYBS-18; 13-18: ZYBS-19; 19-24: ZYBS-23


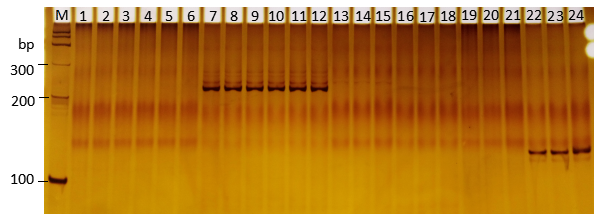


**C** 1-6: ZYBS-24; 7-12: ZYBS-33; 13-18: ZYBS-46; 19-24: ZYBS-47

**
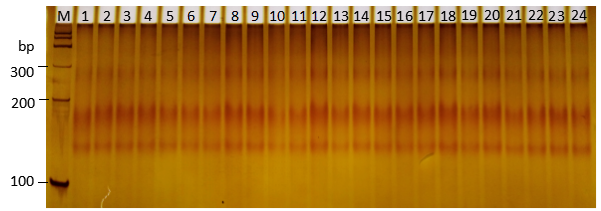
**

**D** 1-6: ZYBS-51; 7-12: ZYBS-52; 13-18: ZYBS-56; 19-24: ZYBS-60

**
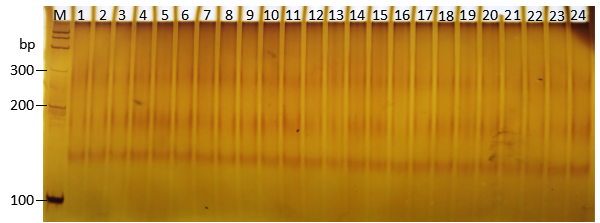
**

**E** 1-6: ZYBS-63; 7-12: ZYBS-66; 13-18: ZYBS-68; 19-24: ZYBS-70

**
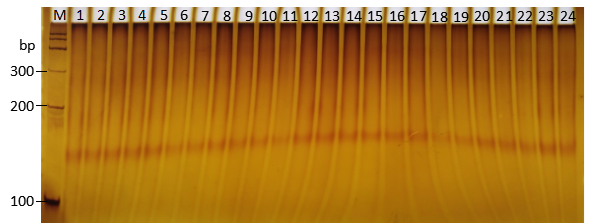
**

**F** 1-6: ZYBS-73; 7-12: ZYBS-84; 13-18: ZYBS-86; 19-24: ZYBS-90

**
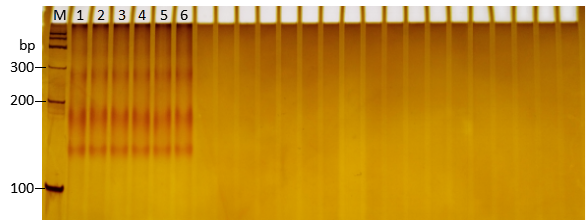
**

**G** 1-6: ZYBS-100

S4 Figure. Cross-amplification results of 25 polymorphic markers in six individuals represented two related species *Phalaenopsis* and *Dendrobium.* For each picture, from 1^st^ to 3^th^, 7^th^ to 9^th^, 13^th^ to 15^th^, 19^th^ to 21^th^ lanes corresponding to the cultivars of *Phalaenopsis,* and the remaining lanes represented the *Dendrobium*.
