# Supplementary material for: De Novo assembly, characterization and development of EST-SSRs from Bletilla striata transcriptomes profiled throughout the whole growing period
Source: PLoS One. 2018 Oct 26;13(10):e0205954. doi: 10.1371/journal.pone.0205954 (PMC6203367; doi:10.1371/journal.pone.0205954)
Supplement: S1 Table — (DOCX) [file pone.0205954.s005.docx]

S1 Table. Landraces information in validation approach of random selected EST-SSR markers

| Landrace | Main phenotypical character | Collection site |
| --- | --- | --- |
| HH | Yellow petal, purple testa | Zheng’an, Guizhou, China  Chishui, Guizhou, China |
| BH | White petal, purple testa |  |
| ZS | Purple petal, purple testa | Hanzhong, Shanxi, China  Bozhou, Anhui, China |
| LS | Purple petal, green testa |  |

Note: All materials are planted in Zunyi, Guizhou, China. And due to the lack of detail source description, the other information of these materials is unknown.
