# Supplementary material for: De Novo assembly, characterization and development of EST-SSRs from Bletilla striata transcriptomes profiled throughout the whole growing period
Source: PLoS One. 2018 Oct 26;13(10):e0205954. doi: 10.1371/journal.pone.0205954 (PMC6203367; doi:10.1371/journal.pone.0205954)
Supplement: S2 Table — (DOCX) [file pone.0205954.s006.docx]

S2 Table. Cultivars information in cross-species validation approach of 25 polymorphic EST-SSR markers

| Cultivars | Collection site | Geographic coordinates | n |
| --- | --- | --- | --- |
| *Phalaenopsis* | Suqian, Jiangsu, China | 33°58′N, 118°16′E | 3 |
| *dendrobium* | Chishui, Guizhou, China | 28°57′N, 105°69′E | 3 |

Note: All materials are planted in Zunyi, Guizhou, China. And due to the lack of detail source description, the other information of these materials is unknown.
