# Supplementary material for: De Novo assembly, characterization and development of EST-SSRs from Bletilla striata transcriptomes profiled throughout the whole growing period
Source: PLoS One. 2018 Oct 26;13(10):e0205954. doi: 10.1371/journal.pone.0205954 (PMC6203367; doi:10.1371/journal.pone.0205954)
Supplement: S3 Table — (DOCX) [file pone.0205954.s007.docx]

S3 Table. Summary of annotation results in different databases of all unigenes

| Database | Unigenes No. | Percentage (%) |
| --- | --- | --- |
| NR | 49,355 | 37.93 |
| NT | 34,751 | 26.70 |
| KO | 20,764 | 15.93 |
| Swiss-Prot | 40,109 | 30.82 |
| PFAM | 40,920 | 30.44 |
| GO | 41,818 | 32.13 |
| KOG | 24,615 | 18.91 |
| Annotated in all databases | 10,098 | 7.76 |
| Annotated in at least one database | 67,494 | 50.86 |
| Total | 130,137 | 100.00 |
